# The genus *Nocardia* as a source of new antimicrobials

**DOI:** 10.1038/s44259-025-00075-6

**Published:** 2025-01-25

**Authors:** Napawit Nonthakaew, Liam K. R. Sharkey, Sacha J. Pidot

**Affiliations:** Department of Microbiology and Immunology, Peter Doherty Institute for Infection and Immunity, University of Melbourne, Melbourne, VIC Australia

**Keywords:** Natural product synthesis, Antimicrobials, Bacteriology

## Abstract

The genus *Nocardia* comprises over 130 species of soil-dwelling actinomycetes, many of which are opportunistic pathogens. Beyond their pathogenicity, *Nocardia* exhibits significant biosynthetic potential, producing an array of diverse antimicrobial secondary metabolites. This review highlights notable examples of these compounds and explores modern approaches to unlocking their untapped biosynthetic potential. As a relatively underexplored genus, *Nocardia* represents a promising source for new antibiotics to combat the growing resistance crisis.

## Introduction to the genus *Nocardia*

*Nocardia* is a genus of gram-positive, high-GC, aerobic, acid-fast bacteria widely distributed in soil and aquatic environments, where they play crucial roles in the decomposition of organic matter. Named after French bacteriologist Edmond Nocard and first isolated in 1888, *Nocardia* species are known for their branching filamentous morphology under the microscope and distinct substrate and aerial mycelia that fragment into coccoid and rod-like forms. As members of the order Mycobacteriales within the phylum Actinomycetota, *Nocardia* are closely related to other mycolic acid-producing bacteria including Mycobacteria and Corynebacteria. The genus *Nocardia* has undergone significant taxonomic revision with the advent of molecular phylogenetic techniques, with many organisms that were once classified as *Nocardia* based primarily on morphologic features assigned to other genera such as *Actinomadura*, *Amycolatopsis*, *Oerskovia*, *Rhodococcus*, and *Rothia*^1^. Currently, 131 species have validly published names, of which at least 54 are known to cause infections in humans^2^. This places the *Nocardia* as number three on the list of genera with the most known pathogenic species^3^.

As opportunistic pathogens, *Nocardia* can cause a range of infections, primarily in immunocompromised individuals, although approximately 40% of all infections occur in immunocompetent patients^4^. Nocardiosis, the clinical manifestation of *Nocardia* infection, can occur in the lungs, skin, and brain, but can disseminate to virtually any organ system. The most clinically relevant species are the *N. asteroides* complex, *N. farcinica* and *N. brasiliensis*, although there appear to be geographic differences in species prevalence^5^. The global incidence of *Nocardia* infections is currently unknown, although cases appear to be increasing in several regions around the world^6,7^. While recent data is hard to come by, cases appeared to triple in the decade between 1998 and 2008 in Canada, while the case-based incidence of nocardiosis in northern Australia was 2.02 per 10,000 over a similar time period^8,9^. Treatment is dependent on infection site and immune status, but can be long-term (6–12 months) and often involves a combination of antibacterial agents^2^.

Apart from their pathogenic potential, *Nocardia* species are also notable for their ability to produce complex bioactive secondary metabolites with antibiotic, antifungal, and immunosuppressive properties^10,11^. The production of a bacterial secondary metabolite is commonly encoded by genes that are clustered together on the bacterial genome in a biosynthetic gene cluster (BGC). Like their relatives in the genus *Streptomyces*, genome sequencing efforts have revealed that the *Nocardia* typically harbour numerous BGCs, many of which remain transcriptionally “silent” under standard laboratory conditions, suggesting an untapped reservoir of novel bioactive compounds^12^. These findings have attracted significant interest in the field of natural product discovery and biotechnology, with hopes that the *Nocardia* can be used as a source of next-generation antimicrobials.

In this review we have selected several known antibiotics from *Nocardia* to illustrate their potential as producers of bioactive metabolites, discussing their discovery and subsequent investigations. We also discuss the biosynthetic potential of this genus and explore recent advances that could be used to unlock the production of antimicrobials.

## Known antimicrobials from *Nocardia*

Antibiotics were first reported to be produced by *Nocardia* in 1947, with the observation that a contaminant (later identified as *N. coeliaca*) inhibited the growth of a *Mycobacterium tuberculosis* culture^13^. However, despite this early report and several more in the years that followed, as a group of secondary metabolite producers the *Nocardia* have received relatively limited attention compared to their better-studied cousins in the genus *Streptomyces*. This is particularly evident when searching through databases of known compounds, such as the Dictionary of Natural Products^14^, where a search for “Streptomyces” yields > 10,000 compounds, while a search for “Nocardia” gives only 340 results. Of these 340 compounds, 239 are listed as having any bioactivity, with 180 listed specifically as having antimicrobial activity. Despite this small number, the *Nocardia* produce a range of antimicrobials, several with interesting and unique modes of action. A selection of these antimicrobials and their properties are outlined below.

### Nargenicin

**Fig. 1:**
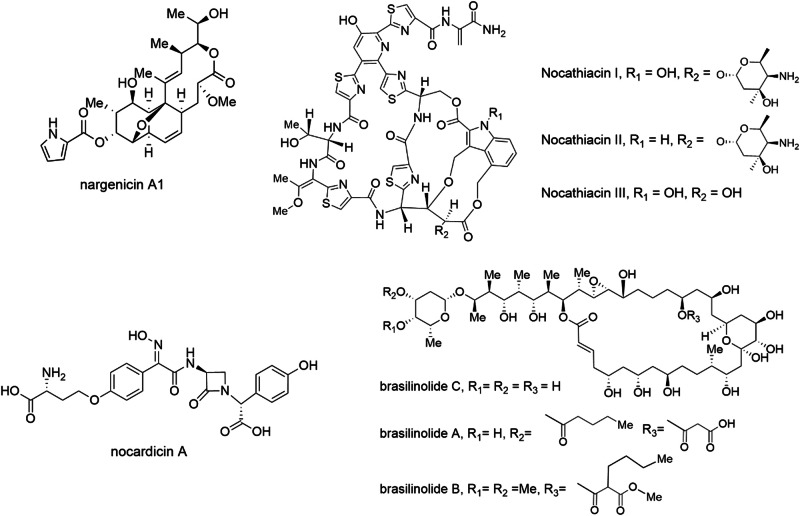
Structures of *Nocardia-*derived antimicrobials discussed in the text. These antimicrobials are examples of the diverse compound structures and classes that have been isolated from *Nocardia* species.

Of the *Nocardia*-produced antibiotics identified to date, the nargenicins are among the most interesting both in terms of their activity and bacterial target. Nargenicin (known originally as CP-47,444) was first discovered in the early 1980s from a culture of *Nocardia argentinensis*^15^. A narrow-spectrum antibiotic, nargenicin A1 (Fig. 1) is highly active against *Staphylococcus aureus*, including against methicillin-resistant isolates in vitro and in a mouse thigh model, and mycobacteria^16–18^, yet lacks activity against related gram-positive bacteria (i.e. *Streptococcus pneumoniae* and *Bacillus subtilis*) and gram-negative bacteria^18,19^. This narrow spectrum of activity, at a time when broad-spectrum compounds were preferred, presumably led the nargenicins to fall out of favour and they did not receive significant attention for another 30+ years. The recent rise of antibiotic-resistant bacteria and an improved understanding of the importance of microbiota in health and disease has spurred renewed interest in these narrow-spectrum compounds. A new generation of synthetic nargenicin derivatives made by Merck scientists showed that nargenicin A1 could be modified to improve targeting to *S. pneumoniae*, but this came at the cost of increasing *S. aureus* minimum inhibitory concentrations (MICs)^17^. The molecular target of the nargenicins was unknown until 2015 when it was shown to inhibit DNA replication by binding to DnaE (also known as DnaE1 or PolC), the alpha subunit of DNA polymerase III^18^. Further work showed that nargenicin binding to DnaE is DNA dependent and that residues involved in nargenicin binding in *S. aureus, M. tuberculosis*, and *E. coli* are conserved, but it is the polymerase’s affinity for DNA (e.g. *S. aureus* > *M. tuberculosis* > *E. coli*) that is primarily responsible for the differential activity^20^. Although the nargenicins and related molecules such as streptoseomycin^21^ have yet to progress to clinical trials, they remain an interesting group of narrow-spectrum compounds with development potential.

### Nocathiacins

The ribosomally synthesised nocathiacins are a group of structurally related thiazolyl cyclic peptides with similarity to thiostrepton (Fig. 1) and originally isolated from *Nocardia* sp. WW-12651^22^. First discovered in the early 2000s at Bristol-Myers Squibb (BMS) laboratories, these molecules were shown to have activity in the low ng/mL range against several gram-positive pathogens including methicillin-resistant *S. aureus*, multi-drug resistant *Enterococcus faecium* and penicillin-resistant *Streptococcus pneumoniae* in vitro, and were shown to be active in vivo against *S. aureus* in a mouse infection model^22^. Despite their potent activity, the nocathiacins suffered from unfavourable solubility, which led to the development of semi-synthetic derivatives by BMS (and later Merck). Several derivatives with improved solubility were active in vitro against *M. tuberculosis*, *Clostridiodes difficile* (also in an in vivo model) and a limited range of gram-negative bacteria including *Legionella pneumophila* and *Mycoplasma pneumoniae*^23–25^. Like thiostrepton, the nocathiacins target protein translation, binding to the L11 protein of the bacterial ribosome (encoded by *rplK*)^23^. However, unlike many other antibiotics that target the ribosome, the nocathiacins were found to be bactericidal against *S. aureus*^23^. Despite their favourable antimicrobial activity, clinical trials of the nocathiacins and their derivatives have not yet been performed. However, recent work may have renewed interest in thiazolyl cyclic peptide antibiotics, as a derivative of the cyclic thiazolyl GE2270A (known as LFF571) showed non-inferiority to vancomycin in a phase II clinical trial for the treatment of *C. difficile* associated disease^26^. In addition, other recent work on the nocathiacins and their derivatives showed that they also have nanomolar MICs against *Plasmodium falciparum*^27^ and that there may be potential for their use as anticancer agents, as they bind strongly to the nuclease processing site of microRNA-18a (an oncogenic non-coding RNA), which was shown to promote apoptosis in a prostate cancer cell line^28^. Taken together these studies suggest there may be a bright future for the nocathiacins, and thiazolyl peptides in general, as new antimicrobials.

### Nocardicins

Nocardicins A and B are β-lactam antibiotics originally identified from *Nocardia uniformis* subsp *tsuyamanensis* at the Fujisawa pharmaceutical company in the mid-1970s^29^. These molecules were found to have an unusual monocyclic structure, making them the first members of the monobactam sub-class of β-lactams^30^. Early work showed that nocardicin A, in particular, was active in vitro against select gram-negative pathogens, including *Pseudomonas aeruginosa*, *Neisseria* and several *Proteus* species in the low µg/mL range, but inactive against gram-positive bacteria^31^. Interestingly, these MICs were found to be significantly influenced by media components, with sodium chloride and several amino acids antagonising nocardicin A activity^32^. The narrow spectrum of activity against gram-negative aerobic bacilli and lack of activity against gram-positive bacteria is a common feature of monobactams, fitting the structure of nocardicin A^33^. In spite of its relatively modest in vitro activity, nocardicin A progressed to in vivo studies showing activity better than carbenicillin in mice infected with *Pseudomonas aeruginosa* or *Proteus mirabilis*, including against β-lactamase producing strains^34^. The nocardicin A activity spectrum, as well as several unusual structural features, led to many studies to identify its biosynthetic origins, culminating in the discovery of the non-ribosomal peptide synthetase-based pathway from genomic data in the early 2000’s^35^. While the nocardicins have also not progressed into clinical development, the past decade has seen a resurgence in studies on monobactams, primarily due to their resistance to β-lactamases and effectiveness against multidrug-resistant bacteria^33^.

### Brasilinolides

The brasilinolides are a group of antifungal and immunosuppressive 32-membered, structurally unique macrolides. First identified from *Nocardia brasiliensis* IFM0406 (now *Nocardia terpenica*) in the mid-1990s, brasilinolide A (Fig. 1) was found to have antifungal activity specifically against *Aspergillus niger*, but not against other filamentous fungi^36^. Brasilinolide A also exhibited potent immunosuppressive activity with very low cytotoxicity^36^. Subsequently, two natural brasilinolide derivatives (B and C) were identified, with brasilinolide B found to have activity against a much broader range of filamentous fungi and yeasts, albeit with decreased immunosuppressive activity, while brasilinolide C showed no immunosuppressive activity, but exhibited cytotoxicity against murine lymphoma cells^37,38^. This range of activities is thought to be related to modifications of the 2-deoxy-l-fucose moiety, as well as at the C23 hydroxyl position of the various brasilinolide derivatives (Fig. 1)^37,38^. Unfortunately, the mechanism of action of the brasilinolides as antifungals, immunosuppressives or anticancer compounds is still unknown and this may be related to the limited supply of these molecules, as synthetic efforts have not yet managed to provide a route to the final compounds leaving the producing *Nocardia* as the only source^39^. More recent studies have identified the biosynthetic pathway for the brasilinolides, including detailed studies on the production of the important 2-deoxy-l-fucose moiety, providing a pathway for future engineering efforts to develop new brasilinolides with improved properties or activities^40^.

## Potential for future antibiotic discovery from the genus *Nocardia*

As can be seen above, the *Nocardia* are capable of producing a range of antimicrobials with diverse structures and bioactivities. However, the rate of compound discovery from the *Nocardia* clearly lags behind other organisms, such as *Streptomyces*. This is not because *Nocardia* lacks a diversity of genes for producing antimicrobials; a bioinformatic analysis of ~170,000 sequenced bacterial genomes revealed that *Nocardia* is among the top 20 taxa with the highest biosynthetic potential^41^. On average, *Nocardia* genomes harbour between 20 and 40 BGCs that cover all the major secondary metabolite classes^12^, which correlates very well with what is known about the BGC number in Streptomycete genomes^42^. What is perhaps more important than the sheer number of BGCs per genome is that the majority of BGCs encoded in *Nocardia* genomes have low similarity and limited homology to other BGCs with a known product and to BGCs in other actinomycetes, suggesting the potential for the discovery of many new chemical scaffolds^12^.

A key question, then, is how to unlock the unique biosynthetic potential of the *Nocardia*? Given that there is a relative paucity of compounds isolated from *Nocardia* compared to *Streptomyces*, one could argue that this is simply because we have not screened the same number of *Nocardia* species for antibiotic production. On the surface, this may be one explanation, yet we have no real idea of how many *Nocardia* isolates may have been screened in bioactive metabolite discovery programs over the years. However, a brief look at the number of *Streptomyces* species with valid names reveals 733 species as opposed to 131 *Nocardia*, suggesting that many more samples have been investigated for the presence of *Streptomyces* and therefore the number screened is likely to be far in excess of the number of *Nocardia* screened.

**Fig. 2:**
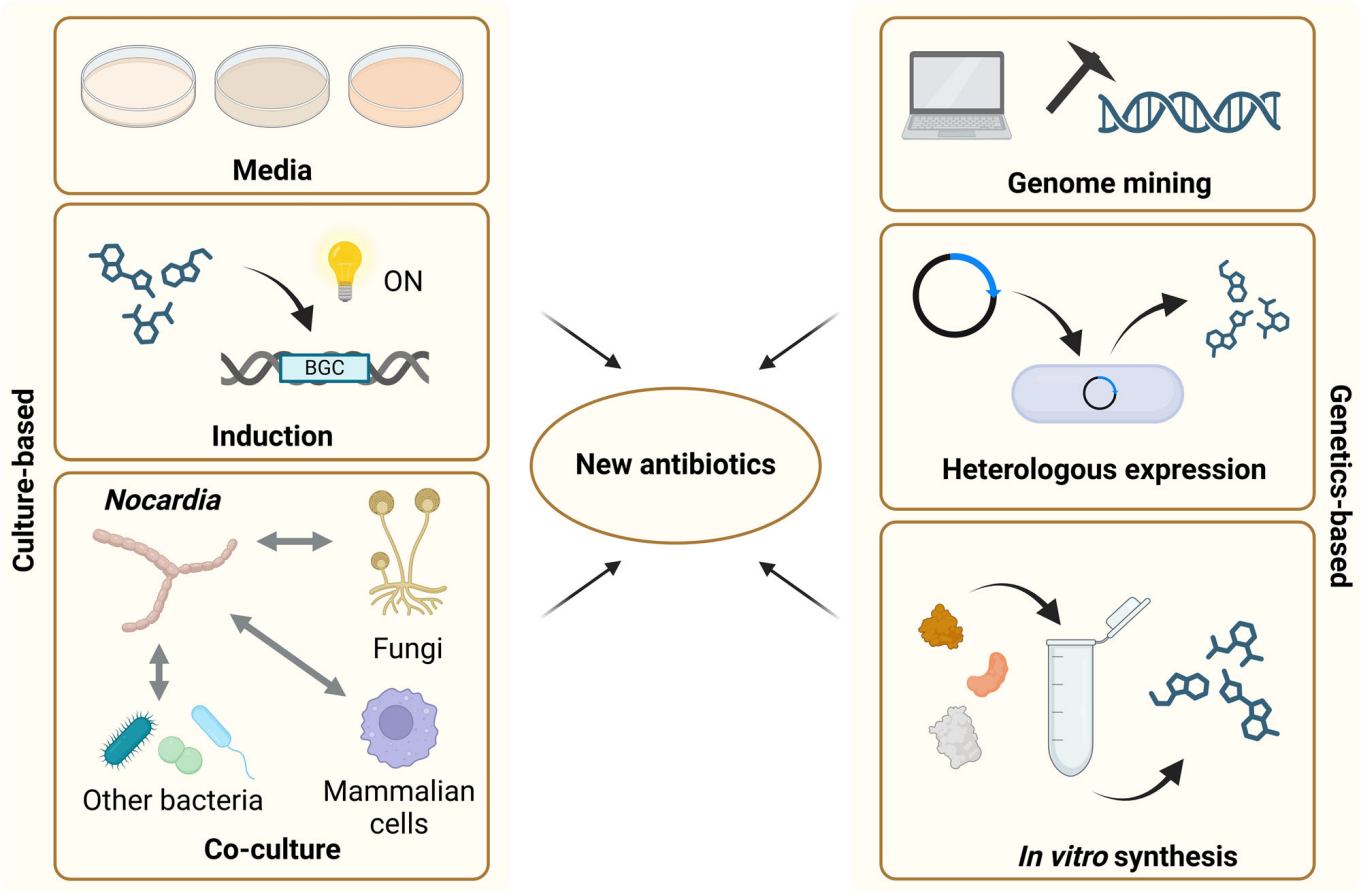
Overview of approaches to elicit new antibiotics from *Nocardia* species. Figure was generated using Biorender.com.

While simply improving the numbers game is one strategy that may well reveal new *Nocardia* antibiotics, the issue of BGCs remaining silent under standard culture conditions is a significant problem^43^. Therefore, a combined approach to antimicrobial discovery in the *Nocardia* utilising a range of modern techniques is necessary to access the full complement of encoded secondary metabolites. We present below a selection of techniques, moving from traditional culture-based techniques to more recent genomic and synthetic biology methods (Fig. 2), with the potential to coax hidden metabolites out of the *Nocardia*.

### Culture-based methods

Culturing microbes on solid or liquid media in the laboratory, performing bioassays, then extracting their secondary metabolites and performing chromatographic analysis has been the standard pathway for antimicrobial discovery since the development of the Waksman platform^44^. However, standard cultivation methods may have limited the number of *Nocardia* species that have been isolated in previous screening campaigns, as Streptomycetes predominate in soil samples^45^. Recent developments using microbial diffusion chambers, where environmental bacteria are trapped but allowed access to nutrients from their natural environment, revealed new *Nocardia* species and identified the neocitreamicins, antibiotics that are active against multidrug-resistant gram-positive bacteria^46,47^.

While standard cultivation methods have helped to identify a range of antimicrobials, the use of standard media types appears to only elicit a small portion of the overall encoded chemical diversity in any one strain^48^. As such, several groups have developed innovative approaches involving the application of stressors to potential antibiotic-producing organisms, to force bacteria to produce new metabolites. One such approach is the use of chemical inducers at low concentrations to simulate environmental conditions. As the *Nocardia* are primarily soil-dwelling bacteria, their natural habitat involves competition with the many other microbes in this environment for scarce resources. Presumably, this means that they may produce antimicrobials under such conditions to protect their territory and maintain their ecological niche. Several groups have used sub-MIC levels of various antibiotics and other natural products of microbial origin to successfully induce the production of new secondary metabolites from actinomycetes^49,50^. In the soil environment, it is not only microbes that send chemical signals; plants also exude a range of compounds from their roots and several *Nocardia* species have been identified from the plant rhizosphere^51,52^. For *Streptomyces*, studies have shown that they can promote plant health through the suppression of disease-causing microorganisms and that they respond to plant-produced metabolites, such as indole-3-acetic acid^53^. Sterols, a subgroup of the steroids and an important component of many eukaryotic cell membranes, are also abundant in soil environments and have been used to induce secondary metabolism in *Streptomyces*^54^. The examples provided here show that induction approaches can elicit the production of novel secondary metabolites, however, these approaches have been relatively poorly explored in the *Nocardia*.

A separate approach that could be likened to an in vivo version of chemical induction is co-culturing. While co-culturing can be performed with any microorganism, in particular, co-culturing approaches with mycolic acid-containing bacteria, such as *Tsukamurella* or *Corynebacterium*, have been shown to induce the production of cryptic metabolites in actinomycetes. Studies have suggested that physical contact between the bacteria is a requirement for induction, although the molecular mechanism remains unclear^55^. As the *Nocardia* are mycolic acid-containing bacteria themselves, whether or not such a system would induce secondary metabolism in these organisms is also uncertain. Co-culture with eukaryotic cells has also been tested as a mechanism for compound induction in actinomycetes. Co-culture of *Streptomyces* and various fungi has revealed a range of new metabolites^56^, yet this approach has not been tested extensively with other actinomycetes. Given that the *Nocardia* are opportunistic pathogens, several groups have investigated the potential for secondary metabolite elicitation when these bacteria are grown in the presence of mammalian cells. Although still in the early stages of discovery, this approach has been shown to induce the production of several previously unknown compounds from a range of *Nocardia* species, posing interesting questions regarding a potential role for secondary metabolites in the pathogenesis of nocardiosis^57^.

Although both induction and co-culture methods have proven to be useful for the elicitation of new secondary metabolites in several actinomycetes, a downside to these (and other) culture-based approaches is that the “brute force” nature of these experiments requires a large combinatorial increase in the number of cultures to be grown and assayed as numbers of induction compounds or co-culture partner organisms increase. While this is feasible for laboratories with access to appropriate robotic systems, the time-consuming nature of these experiments may make them impractical for smaller laboratories. Perhaps the best use of these approaches, to limit the required amount of screening, is to combine these with more modern genetic analyses, as outlined below.

### Genetics-based approaches

Genome mining, or the investigation of DNA sequence data for the presence of secondary metabolite BGCs, has emerged as a powerful strategy for identifying novel biosynthetic pathways and their products. Advances in protein prediction algorithms and our understanding of biosynthetic logic for the majority of secondary metabolite structural classes allowed the development of bioinformatic tools (e.g. antiSMASH^58^ or PRISM^59^) to analyse bacterial genomes and identify and prioritise promising BGCs for experimental characterization. These in silico approaches have revealed numerous cryptic gene clusters in the *Nocardia* (as mentioned above)^12,41^, suggesting a vast reservoir of undiscovered bioactive compounds. However, merely identifying BGCs is not sufficient to reveal new chemistry; the cognate metabolites must be produced to effectively link pathways to products. While the culture-based methods outlined above provide an essentially random approach to inducing secondary metabolism, synthetic biology and genetic engineering approaches allow focused efforts on individual BGCs. Several such approaches have already been applied successfully to uncover hidden biosynthetic pathways and their associated metabolites.

For example, genome mining combined with gene inactivation by homologous recombination was used to identify the biosynthetic pathway for the antibiotic nargenicin in multiple *Nocardia* species^16,60^. Interestingly, inactivation of the nargenicin pathway in *Nocardia* sp. CS682 by homologous recombination-based deletion of the entire polyketide synthase-encoding region was found to expand the pool of available acetyl CoA and malonyl CoA precursors, redirecting metabolic flux and allowing the production of a new tetrahydroxynaphthalene that was not produced by the parent strain^61^. Thus, the inactivation of one pathway can allow the activation of other pathways, providing opportunities to discover previously unknown metabolites. Another successful approach in the *Nocardia* has been to combine genome mining with transposon mutagenesis to overcome difficulties with targeted gene deletions. Such a strategy was used to identify the biosynthetic pathway for the new molecule terpenomycin from a human pathogenic *Nocardia* isolate^62^. Structurally related to bafilomycin, terpenomycin has both significant cytotoxic and antifungal activity, particularly against filamentous fungi^62^.

Several CRISPR-Cas genome editing tools exist for *Streptomyces*, and while there are scattered examples of their use in other actinomycetes, there has not been large-scale uptake or use for editing *Nocardia* genomes. However, two recent studies have successfully shown that CRISPR tools developed for *Streptomyces* can be successfully used to inactivate genes in *Nocardia* species^63,64^. In a nargenicin pathway deletion mutant of *Nocardia* sp. CS682, overexpression of a pathway regulator led to the production of anti-Staphylococcal isofuranonaphthoquinone, NOC-IBR2, with CRISPR-Cas editing used to then delete the regulator and confirm its involvement in the production of this new molecule^63^. Further development and use of efficient CRISPR-Cas genome editing systems in the *Nocardia* will have a significant impact on our ability to identify new antimicrobials from these bacteria.

Beyond deletion strategies, heterologous expression has proven valuable for characterizing BGCs. This approach involves the transfer and expression of target BGCs in well-characterized host organisms, usually *Streptomyces* species. A notable example is the successful activation of a silent type II PKS BGC from *Nocardia brasiliensis* using Transformation-Associated Recombination (TAR) cloning and heterologous expression in *Streptomyces coelicolor*^65^. This resulted in the production of brasiliquinone B, an anti-*Staphylococcus* antibiotic demonstrating the effectiveness of heterologous systems in awakening silent BGCs from *Nocardia* species.

There have also been great advances in the use of in vitro expression systems to produce bacterial secondary metabolites. These cell-free systems use whole-cell lysates or purified proteins along with biosynthetic precursors to produce metabolites in vitro. Several toolkits have been developed specifically for the expression and investigation of *Streptomyces* BGCs^66^ and they have also been used to reconstruct a polyketide biosynthetic pathway that was conserved among several pathogenic *Nocardia* strains^67^. This particular system involved the reconstitution of the complete polyketide synthase pathway following expression and purification of the individual proteins from *E. coli* and subsequent in vitro synthesis to produce the molecule known as nocardiosis-associated polyketide (NOCAP)^67^. The authors were also able to show the production of the NOCAP molecule in *E. coli* without subsequent protein purification, providing a pathway to “deorphanize” BGCs with unknown products in a more rapid fashion^67^.

The use of genome sequence data in isolation and subsequent manual searches through identified BGCs is a time-consuming process. Recent developments in artificial intelligence (AI) provide hope that these more mundane tasks can be accelerated in order to more rapidly find bioactive molecules. There have been several efforts to predict chemical structures from BGC sequence data, and while these algorithms can usually predict a portion of the molecular structure correctly, they often struggle to predict the final product due to enzymatic tailoring reactions that occur in many biosynthetic pathways. These programs, however, can help to prioritise BGCs that may have unusual structural motifs or that do not feature prominently in the realm of known secondary metabolites. Likewise, AI models are also being developed to predict the biological activity of unknown compounds directly from BGC information and to integrate genomic and metabolomic datasets for rapid dereplication and prioritisation of unknowns^68^. However, as with all AI models, a key consideration is the amount of available data that can be used to develop the model and test hypotheses. As future efforts to identify new secondary metabolites from the *Nocardia* and other actinomycetes progress, this will reinforce the strength and predictive ability of our currently available models.

## Conclusions

Despite their ability to produce antimicrobials, the *Nocardia* have not been as well studied as many of their actinobacterial cousins. While it is clear that they have the biosynthetic potential to make a variety of bioactive metabolites, standard cultivation techniques do not appear to be sufficient to unlock the majority of the metabolites encoded within *Nocardia* genomes. The relatively slow growth rates, complex morphological development and minimal suite of genetic tools complicate the identification of new antimicrobials from these bacteria. Additionally, while there are several *Nocardia* species that can be handled at biosafety level 1, many are classified as opportunistic pathogens, necessitating appropriate biosafety containment measures. Despite these challenges, there have been significant recent advances in genetic tools leading to the identification of new molecules (e.g. CRISPR-Cas to identify NOC-IBR2^63^; transposon mutagenesis to identify terpenomycin and its biosynthetic pathway^62^), as well as synthetic biology methods to unveil hidden compounds (such as NOCAP^67^).

As we have described here, the antimicrobial potential of the *Nocardia* remains largely untapped. While challenges in terms of discovery still remain, the unique structural features and biological activities of *Nocardia* metabolites discovered so far provide evidence that there may yet be many new antibiotics to come from these organisms. As antibiotic resistance continues to pose a global health threat, the exploration of underutilised taxa such as *Nocardia* becomes increasingly important for discovering novel antimicrobial compounds.

## Data Availability

No datasets were generated or analysed during the current study.
